# Radiofrequency Ablation of Indeterminate Thyroid Nodules: The First North American Comparative Analysis

**DOI:** 10.3390/ijms231911493

**Published:** 2022-09-29

**Authors:** Peter P. Issa, Mahmoud Omar, Chad P. Issa, Yusef Buti, Mohammad Hussein, Mohamed Aboueisha, Ali Abdelhady, Mohamed Shama, Grace S. Lee, Eman Toraih, Emad Kandil

**Affiliations:** 1 School of Medicine, Louisiana State University Health Sciences Center, New Orleans, LA 70112, USA; 2 Department of Surgery, School of Medicine, Tulane University, New Orleans, LA 70112, USA; 3 Department of Surgery, United Health Services Southern California Medical Education Consortium, Temecula Valley Hospital, Temecula, CA 92592, USA; 4 Department of Surgery, School of Medicine, Yale University, New Haven, CT 06520, USA; 5 Genetics Unit, Department of Histology and Cell Biology, Faculty of Medicine, Suez Canal University, Ismailia 41522, Egypt

**Keywords:** radiofrequency ablation, RFA, indeterminate thyroid nodules, thyroid, Bethesda III, Bethesda IV

## Abstract

Thyroid nodules can be classified as benign, malignant, or indeterminate, the latter of which make up 10–30% of nodules. Radiofrequency ablation (RFA) has become an attractive and promising therapy for the treatment of benign thyroid nodules. However, few studies have investigated the safety and efficacy of RFA for the management of indeterminate thyroid nodules. In this study, 178 patients with thyroid nodules diagnosed as benign (Bethesda II) or indeterminate (Bethesda III/IV) by preoperative cytopathological analysis were included. Patients in the benign and indeterminate cohorts had similar thyroid nodule volume reduction rates at 65.60% and 64.20%, respectively (*p* = 0.68). The two groups had similar nodular regrowth rates, at 11.2% for benign nodules and 9.40% for indeterminate nodules (*p* = 0.72). A total of three cases of transient dysphonia were reported. RFA of indeterminate thyroid nodules was comparable to that of benign thyroid nodules in all parameters of interest, including volume reduction rate. To our best knowledge, our work is the first North American analysis comparing benign and indeterminate thyroid nodules and suggests RFA to be a promising modality for the management of indeterminate thyroid nodules.

## 1. Introduction

Thyroid nodules can be classified as benign, malignant, or indeterminate, the latter of which make up 10–30% of nodules and often display atypical morphological changes, such as Hürthle cells, follicular neoplastic growth, or hypercellularity [1,2]. These changes attribute the indeterminate nodule subclass to a relative risk of that between benign and cancerous nodules and, consequently, has blurred the appropriate aggressiveness of treatment. Current American Thyroid Association (ATA) guidelines recommend lobectomies, hemithyroidectomies, and even total thyroidectomies all as first-line forms of treatment [3,4,5]. While surgery has been largely successful with rates as high as 91%, it is not without risk, including hypocalcemia, hypothyroidism, and recurrent laryngeal nerve injury [6].

Radiofrequency ablation (RFA) has become an attractive and promising therapy for treating thyroid nodules whereby surgeons can strategically thermally ablate lesions. RFA of thyroid nodules is a minimally invasive treatment option that initially began as an alternative treatment modality for poor surgical candidates [7]. Recent studies have shown RFA to be excellent from both safety and efficacy standpoints, with patients achieving between 70% and 90% volume reduction rates (VRR) [7,8,9,10,11]. Though an attractive treatment modality, the current ATA guidelines recommend RFA only for the management of benign thyroid nodules, necessitating the treatment of both indeterminate nodules (10–30% of all nodules) and malignant nodules (5% of all nodules) with surgery [3]. International literature, however, suggests RFA to be promising beyond its typical treatment of benign nodules. For example, a meta-analysis of 1822 papillary thyroid microcarcinomas found a 79% tumor disappearance rate using RFA [12].

A paucity of literature has investigated RFA of indeterminate nodules. A single-institution case series of 10 patients found a 99.5% volume reduction rate utilizing RFA for the treatment of follicular neoplasms [13]. Likely due to the novelty of RFA treatment, a United States-based analysis of indeterminate thyroid nodule RFA has never been conducted, leaving the effectiveness of RFA on a significant portion of nodules unknown. To our best knowledge, this is the first United States-based comparative analysis investigating the safety and efficacy of RFA of indeterminate thyroid nodules with benign nodules.

## 2. Results

### 2.1. Study Population

A total of 178 nodules preoperatively determined to be Bethesda II-IV underwent RFA, of which 125 were benign and 53 were indeterminate. The median age of patients with benign and indeterminate nodules were 65 years (interquartile range (IQR): 52.5–70) and 63 years (IQR: 56–68), respectively (*p* = 0.49). The benign patient cohort was predominately female (78.4% versus 56.6%; *p* = 0.003). There was no difference with respect to patient race between the two cohorts (*p* = 0.82). Median BMI did not differ between the benign (median: 29.8 kg/m^2^, IQR: 26.4–34.3) and the indeterminate (median: 30.5 kg/m^2^, IQR: 27.5–34.0) groups (*p* = 0.38). Baseline characteristics of patients and their treated thyroid nodules are depicted in Table 1.

The two groups did not differ with respect to baseline sonographic features. Benign and indeterminate thyroid nodule patient cohorts had similar maximum nodular diameters (median: 2.3, IQR: 1.4–3.7; versus median: 2.5, IQR: 1.5–4.3; *p* = 0.47) as well as similar baseline volumes (median: 1.7, IQR: 1.1–2.7; median: 1.9, IQR: 1.1–3.2; *p* = 0.45). Furthermore, nodule composition (*p* = 0.31), echogenicity (*p* = 0.74), vascularity (*p* = 0.54), elastography (*p* = 0.27), and calcification classification (*p* = 0.25) did not differ between benign and indeterminate nodules. With respect to function, the benign and indeterminate cohorts had median pre-operative thyroid-stimulating hormone (TSH) values of 1.2 uIU/mL (IQR: 0.8–1.8) and 1.1 uIU/mL (IQR: 0.7–1.8), respectively (*p* = 0.58).

### 2.2. Volume Reduction Rates

**Figure 1:**
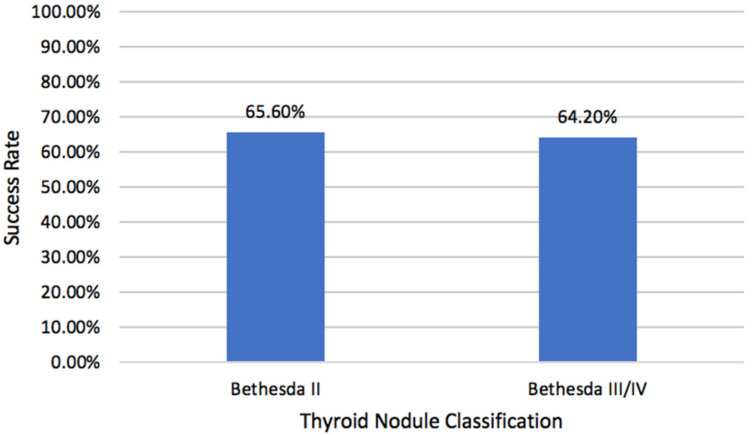
Success rates of benign and indeterminate thyroid nodules treated by RFA. Success was a volume reduction rate of ≥50%.

The efficacy of RFA with respect to volume reduction was assessed. Final VRR within patient 1-year follow-up is shown in Figure 1. The overall final VRR for all patients was 62.4% (IQR: 37.4–81.8). VRR rates of the benign and indeterminate nodule cohorts were 62.1% (IQR: 37.1–80.7) and 69.2% (IQR: 36.4–84.3), respectively (*p* = 0.68). A total of 116 patients (65.2%) achieved operative success (final VRR ≥ 50%). Additionally, 65.6% (82/125) of patients with benign nodules and 64.2% (34/53) of patients with indeterminate nodules achieved operative success (*p* = 0.86).

### 2.3. Complications

A total of three complications were reported. All complications were cases of transient dysphonia (passive return of voice within 3 months), experienced by a pair of patients in the benign group and a single patient in the indeterminate group. Neck pain, a minor side effect, was reported in the case of two patients in the benign cohort as well. No patients presented with hematoma post-ablation.

### 2.4. Nodular Regrowth

**Figure 2:**
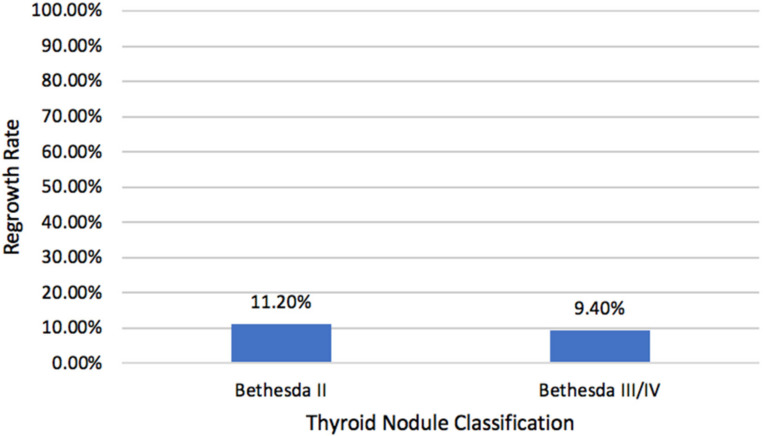
Regrowth rates of benign and indeterminate thyroid nodules treated by RFA. Nodular regrowth was defined as a nodule, which was assessed by ultrasound to be of greater volume postoperatively than pre-operatively.

Since one characteristic of cancerous cells includes sustained and rapid proliferation, we analyzed the regrowth rates of nodules (Figure 2). While 116 patients (65.2%) achieved success (final VRR ≥ 50%), only 19 (10.7%) nodules regrew to a size larger than was determined on preoperative assessment. When sub-grouped by their Bethesda classification, the two groups had similar regrowth rates. Benign nodules grew back with a frequency of 11.2%, while indeterminate nodules grew back with a frequency of 9.4% (*p* = 0.72). Of the nodules that regrew, most did so within the first month (42.1%, 8/19).

## 3. Discussion

RFA has been around for several decades, with utility in tumor ablation in solid organs such as the liver, kidney, bone, lung, and adrenals [14]. In the United States, RFA was only recently FDA-approved as a treatment modality for thyroid nodules, which are deemed twice benign on preoperative fine-needle aspiration and/or core-needle biopsy. With that, RFA has seen tremendous success with volume reduction rates as high as 70–90% with minimal reports of complication [15,16,17]. Internationally, RFA has demonstrated success in the treatment of papillary thyroid microcarcinomas and recurrent thyroid cancers [12]. Few studies have investigated the efficacy and safety of indeterminate nodule RFA in the United States. We found that the two cohorts had similar success rates and regrowth rates. To our best knowledge, our work is the first North American analysis comparing the treatment of benign and indeterminate thyroid nodules by RFA.

Indeterminate nodules are those classified as Bethesda III/IV, corresponding to follicular adenoma, follicular hyperplasia, and Hürthle cell hyperplasia, among others. Bethesda III nodules are ascribed a low risk of malignancy, between 5 and 15%. Bethesda IV nodules are associated with a moderate risk of malignancy, between 15 and 30% [18,19,20]. In accordance with the American Association of Clinical Endocrinologists (AACE), Associazione Medici Endocrinologi (AME), and European Thyroid Association (ETA) guidelines, this cohort of indeterminate nodules are recommended to undergo surgery, except for instances with favorable sonographic, cytologic, and clinical data, which may allow for consideration of active surveillance management (the close monitoring of the patient with yearly or twice-yearly imaging studies to assess tumor progression) [20]. Currently, the ATA and Korean guidelines do not recommend RFA for indeterminate thyroid nodule treatment [3,7]. Though surgery has been the longstanding mainstay treatment, it carries significantly more risk of complication than RFA (6.0% versus 1.0%, *p* = 0.002) [21] and necessitates longer operative times as well (62.9 min versus 8.0 min; *p* < 0.001) [22]. Conversely, RFA is a minimally invasive office-based procedure that obviates the need for general anesthesia and could serve specific patient populations including poor surgical candidates and the elderly [23].

Several works have reported an overall acceptable experience with indeterminate thyroid nodule RFA. Most recently, Lin et al. treated 22 follicular neoplasms and reported a volume reduction of 73.3% at 6–12 months [24]. The authors reported only a single complication of temporary (3 months) vocal cord palsy [24]. A separate work of 10 small (<2 cm) follicular neoplasms were treated by RFA and achieved VRRs of 99.5%, with eight lesions disappearing completely. Importantly, the authors noted no recurrence in the 60 months (5 years) that the patients were followed up [13]. These works suggest that RFA could be used as a treatment modality for indeterminate thyroid nodules. Our work corroborates this notion, finding similar VRRs at 3–12 months and success rates between the two groups.

Several factors contribute to the hesitancy of adopting indeterminate thyroid nodule RFA as a management strategy. First, RFA foregoes the determination of specimen pathology. Unlike traditional thyroidectomy, RFA leaves healthcare teams without important information, leaving patients with considerable anxiety [25]. Another factor is the paucity of data regarding thyroidectomy after RFA. Quantitative assessments of post-RFA thyroidectomy efficacy and safety have been largely unexplored. A small study reporting the treatment of six patients with Bethesda III/IV nodules with RFA successfully treated two nodules that regrew by thyroidectomy. Though only qualitatively assessed, the authors noted no difficulty in the ligation of major vessels, no difference in thyroid parenchyma, and no substantial adhesions [26]. Future studies quantitively assessing outcomes of post-RFA thyroidectomies as well as comparative works (primary thyroidectomy versus post-RFA thyroidectomy) are warranted to scientifically address this potential concern. All three of the above-described works investigating the efficacy of indeterminate thyroid nodule RFA were conducted outside of the United States.

One characteristic of cancerous cells is rapid proliferation. While the primary intent of RFA is to mitigate and resolve compressive symptoms in patients with benign thyroid nodules, insufficient ablation may prompt nodular growth. In a cohort of 206 benign thyroid nodules, the incidence of regrowth following RFA was 12.62% (26/206) [27]. Similarly, a large long-term (5-year follow-up) multicenter study found benign nodular regrowth to occur with 20% frequency [28]. These results are comparable to our work, which showed 11.2% and 9.4% regrowth rates in benign and indeterminate thyroid nodules, respectively.

The decision to undergo RFA as opposed to thyroidectomy is especially important in patients with indeterminate thyroid nodules as the literature is still in its infancy. Though thyroid lobectomy is the primary treatment option for indeterminate or symptomatic benign nodules, the risk of complication with thyroid resection can be as high as 10% [29]. Conversely, RFA has an exceptional safety profile with a multi-institutional study of 1459 nodules reporting only a 3.3% overall complication rate [30]. In our study, the overall complication rate was 1.69%, with no reports of hematoma and only three incidences of transient dysphonia. This is consistent with the large multi-institutional study aforementioned, which reported voice changes as the most common complication with a frequency of 1.03% [30]. None of our patients had nodular rupture. In addition, RFA obviates the need for general anesthesia, which is more suitable and attractive for elderly patients or those with significant comorbidity. RFA can be performed in a clinic, removing the cost of an operating room. From a financial perspective, RFA of indeterminate nodules could potentially be more cost-friendly than thyroidectomy, as is the case for both benign [31] and low-risk papillary thyroid carcinomas [32]. Finally, patients undergoing RFA typically only complain of minimally discomforting symptoms, which typically resolve on their own.

## 4. Materials and Methods

### 4.1. Study Design

Upon approval by the Institutional Review Board of Tulane University, we conducted our prospective study to assess RFA of indeterminate and benign thyroid nodules. Our protocol consisted of preoperative fine-needle aspirations as well as comprehensive neck ultrasounds; treatment by RFA; and subsequent follow-up appointments at the 1, 3, 6, and 12 month marks. All operations were performed by fellowship-trained endocrine surgeons (E.K., M.S., and G.L.) between 2018 and 2022. All patients willingly consented to participate in this study.

### 4.2. Recruited Cohort

Preoperative assessment of each nodule included both fine needle aspiration and ultrasound imaging. Pre-operative comprehensive neck ultrasound assessment, using GE Logiq 9 US1 and a 15-MHz linear transducer, determined nodule composition, elastography, echogenicity, vascularity, and dimensions. Two testing platforms, including Afirma Thyroid FNA Analysis (including GEC and GSC; Veracyte Inc., San Francisco, CA, USA) and Interpace ThyGenX/ThyraMIR (Interpace Biosciences, Parsippany, NJ, USA), were utilized to analyze thyroid cytology. Most nodules were examined by fine-needle aspiration biopsy twice, though some nodules which were benign-appearing on ultrasound were biopsied only once. The Bethesda System was utilized for reporting thyroid cytopathology, wherein nodules were classified as (I) nondiagnostic, (II) benign, (III) atypia of undetermined significance, (IV) follicular neoplasm or suspicious for a follicular neoplasm, (V) suspicious for malignancy, and (VI) malignant [18,33].

Thyroid nodules were included only if they (1) were classified by fine needle aspiration as a Bethesda II, III, IV nodule; (2) have yet to be treated (no previous laser ablation or ethanol ablation or radioactive iodine ablation); and (3) attended at least one follow-up visit beyond the 1-month mark. By the nature of the study, Bethesda VI nodules were excluded. Since the ETA/AME/AACE all classify only Bethesda III and IV as indeterminate nodules, we excluded patients with Bethesda V nodules [20].

Beyond preoperative ultrasound and fine needle aspiration, patient demographics, including race, gender, age, and BMI, as well as thyroid function tests were collected. Procedural parameters, including energy, power, impedance, and probe size were recorded. Post-operative nodule size, thyroid function tests, and patient complaints were collected at the aforementioned follow-up times. In light of the COVID-19 pandemic, patient adherence to follow-up appointments was sub-optimal. All patients included in the study had at least a single follow-up at three months or beyond.

### 4.3. Definitions

With regard to ultrasound classification, we emulated the standard terminology and reporting criteria, which have been utilized widely [22,34]. Nodule composition was classified as solid (cystic portion < 10%), cystic (cystic portion > 90%), or mixed. Nodule elasticity was classified as stiff (>80% stiffness), soft (<20%), or mixed. Nodule vascularity was classified on a four-point scale: grade 0 (lacking flow signal), grade 1 (peripheral flow), grade 2 (flow within nodule < 50%), and grade 3 (flow within nodule > 50%).

Nodule volume was calculated as the length × width × height. VRR was calculated as [(V_0_ − V_1_)/V_0_] × 100, where V_0_ signifies the initial nodule volume and V_1_ signifies the post-ablation nodule volume. VRR was utilized to define operative success. Operative success was defined as a VRR of >50% at final follow-up [13]. Nodular regrowth was described as a nodule that was assessed by ultrasound to be of greater volume postoperatively than pre-operatively. VRR, operative success rate, and nodular regrowth rate are expressed as percentages, consistent with other works in the field [35].

### 4.4. RFA Procedure

RFA is the usage of a probe to thermally ablate the thyroid gland [36]. Procedures were conducted in an outpatient operating room with an 18-gauge STARmed (Seoul, Korea) internally cooled electrode with an active tip of either 5 or 7 mm. Prior to RFA initiation, the entirety of the neck was prepared with povidone-iodine swabs, treated with local anesthetic at the puncture site, and oftentimes draped.

RFA commenced most often by the long axis technique (trans-isthmic), though several procedures were performed using a short axis approach. All procedures utilized the moving-shot technique [37]. Rarely, nodules were in the danger triangle (i.e., near the anticipated location of the recurrent laryngeal nerve) in which nodules accordingly were ablated with a lateral to medial approach.

Initially, the probe was inserted into the deepest portion of the nodule and slowly drawn superficially. Initial ablation power was 15 Watts (W), and impedance was allowed to reach at least 200 ohms. Surgical preference in early procedures was to elicit nodular bubbling; however, this emphasis waned, and preference grew for impedance values of at least 200. For most nodules, RFA aimed to attain operative success (>50% VRR) by a single treatment session; however, in cases of especially large nodules, operative preference aired on the side of caution such that a patient may undergo two ablation sessions.

Patient communication was encouraged during the operation to allow for the assessment of laryngeal nerve function. The presence of hoarseness or a change in voice quality immediately halted the procedure.

### 4.5. Postoperative Evaluation

Patients were discharged following a 30 min observation period and flexible laryngoscopy. At follow-up visits, patients were evaluated for their symptoms, recent changes, complications, and biochemical data [36]. Neck ultrasound was completed in the same manner with which it was initially assessed as so as to determine nodule volume.

### 4.6. Statistical Analysis

Statistical analysis was performed using SPSS version 27.0 and SAS 9.4. Characteristics and outcomes of benign (Bethesda II) and indeterminate nodules (Bethesda III and IV) were compared. Categorical variables are expressed with percentages. Continuous variables were reported as median with their respective IQR. Two-sided Chi-square and Mann–Whitney U tests were used. *p*-values below 0.05 were considered significant.

## 5. Conclusions

Overall, our work demonstrated similar success, regrowth, and complication rates between benign and indeterminate thyroid nodule RFA. Moving forward, studies of larger sample sizes and longer follow-up duration could realistically posit RFA as a potential treatment option for indeterminate thyroid nodules.

## Figures and Tables

**Table 1:** Baseline characteristics of thyroid nodules treated with radiofrequency ablation with subgroup analysis based on preprocedural cytological analysis.

Characteristics	Levels	Total	Bethesda III and IV Nodules	Bethesda II Nodules	*p*-Value
Number		178	53	125	
Demographic data					
Age	Median (IQR)	64 (53–69)	63 (56–68)	65 (52.5–70)	0.49
	<55 years	48 (27)	12 (22.6)	36 (28.8)	0.39
	≥55 years	130 (73)	41 (77.4)	89 (71.2)	
Gender	Female	128 (71.9)	30 (56.6)	98 (78.4)	0.003
	Male	50 (28.1)	23 (43.4)	27 (21.6)	
Race	African American	100 (56.2)	28 (52.8)	72 (57.6)	0.82
	White	71 (39.9)	23 (43.4)	48 (38.4)	
BMI	Median (IQR)	30 (26.7–34.3)	30.5 (27.5–34.5)	29.8 (26.4–34.3)	0.38
Baseline sonographic features					
Nodule maximum diameter	Median (IQR)	2.4 (1.5–4)	2.5 (1.5–4.3)	2.3 (1.4–3.7)	0.47
Baseline Volume	Median (IQR)	1.8 (1.1–2.9)	1.9 (1.1–3.2)	1.7 (1.1–2.7)	0.45
Composition	Solid	5 (2.8)	3 (5.7)	2 (1.6)	0.31
	Cystic	157 (88.2)	46 (86.8)	111 (88.8)	
	Mixed	16 (9)	4 (7.5)	12 (9.6)	
Echogenicity	Hypoechoic	33 (21.3)	11 (23.4)	22 (20.4)	0.74
	Isoechoic	121 (78.1)	36 (76.6)	85 (78.7)	
	Hyperechoic	1 (0.6)	0 (0)	1 (0.9)	
Vascularity	Grade 0	11 (7.9)	1 (2.3)	10 (10.3)	0.054
	Grade 1	46 (32.9)	11 (25.6)	35 (36.1)	
	Grade 2	53 (37.9)	23 (53.5)	30 (30.9)	
	Grade 3	30 (21.4)	8 (18.6)	22 (22.7)	
Elastography	Soft	7 (5.3)	4 (10)	3 (3.2)	0.27
	Mixed	96 (72.2)	27 (67.5)	69 (74.2)	
	Stiff	30 (22.6)	9 (22.5)	21 (22.6)	
Calcifications	No Calcifications	91 (58.7)	29 (61.7)	62 (57.4)	0.25
	Microcalcifications	51 (32.9)	12 (25.5)	39 (36.1)	
	Macrocalcifications	13 (8.4)	6 (12.8)	7 (6.5)	
Laboratory data					
Baseline TSH uIU/mL	Median (IQR)	1.3 (0.7–1.9)	1.3 (0.8–1.9)	1.3 (0.7–2)	0.67
Post procedural TSH uIU/mL	Median (IQR)	1.2 (0.7–1.8)	1.1 (0.7–1.8)	1.2 (0.8–1.8)	0.58

Data are presented as number (percentage), or median and interquartile range (IQR). Two-sided Chi-square and Mann–Whitney U tests were used.

## Data Availability

The data are contained within the article.
